# Complex training with blood flow restriction increases power output and bar velocity during half-squat jump: a pilot randomized controlled study

**DOI:** 10.3389/fphys.2024.1368917

**Published:** 2024-05-30

**Authors:** Limingfei Zhou, Yineng Tan, Jianyu Gan, Chunlei Li, Dapeng Bao, Junhong Zhou

**Affiliations:** ^1^ School of Strength and Conditioning Training, Beijing Sport University, Beijing, China; ^2^ Sports Coaching College, Beijing Sport University, Beijing, China; ^3^ China Institute of Sport and Health Science, Beijing Sport University, Beijing, China; ^4^ Hebrew Senior Life Hinda and Arthur Marcus Institute for Aging Research, Harvard Medical School, Boston, MA, United States

**Keywords:** blood flow restriction, complex training, power, jump, strength

## Abstract

**Purpose:**

This study examined the effects of 8-week complex training (CT) with blood flow restriction (BFR) on power output and bar velocity.

**Methods:**

Twenty-six healthy male university athletes (age: 19.40 ± 0.88 years) completed three sessions of CT with BFR (CT_BFRT, n = 13) or CT-only (i.e., control) (n = 13) per week (i.e., 24 sessions in total). Before and immediately after intervention, participants completed power measurement as assessed by one-repetition maximum (1RM) squat, squat jump (SJ), countermovement jump (CMJ), and mean power (MP), peak power (PP), mean bar velocity (Bar-MV), and peak bar velocity (Bar-PV) during the half-squat jump.

**Results:**

Two-way ANOVA models showed significant main effect of time (*p* < 0.001) but not group (*p* > 0.89) or interaction (*p* > 0.37) between group and time on 1RM of the squat, SJ, or CMJ; however, significant interactions were observed in MP (*p* = 0.03, Cohen’s d = 1.39), PP (*p* = 0.03, Cohen’s d = 1.14), Bar-MV (*p* = 0.049, Cohen’s d = 1.26), and Bar-PV (*p* = 0.01, Cohen’s d = 1.56). The *post hoc* analyses revealed that MP, PP, Bar-MV, and Bar-PV after CT with BFRT were significantly greater compared to all the other three conditions (i.e., pre-CT_BFRT, pre- and post-CT-only).

**Conclusion:**

CT with BFR may induce significantly greater improvements in power output and bar velocity during half-squat jump and induce comparable improvements in 1RM of the squat, SJ, and CMJ of males as compared to CT only, suggesting this novel CT with BFR would be a promising strategy to enhance power performance in healthy male university athletes.

## 1 Introduction

Lower-limb power is critical to athletic performance, especially for those with high physical load (e.g., athletes, well-training people) (Haff, Whitley, and Potteiger, 2001; Suchomel, Nimphius, and Stone, 2016). Studies have reported that greater muscle strength and power are associated with better performance (e.g., rate of force development, power of squat) of athletes (Edge et al., 2006; McBride et al., 2009) and lower injury risk of lower limbs (Hewett et al., 1999; van der Horst et al., 2015). Therefore, strategies to optimize power can enhance athletic performance and reduce injury risks in athletes.

One such strategy is complex training (CT), consisting of blocks of high-load resistance training (HLRT) and plyometric training (PT) within one single session (Qiao et al., 2022). CT provides a substantial high-load stimulus (Cormier et al., 2021). Previous studies have shown promise of CT in improving both one repetition maximum (1RM) of strength and jump performance of power in athletes (Cormier et al., 2022). To date, most of the studies using CT primarily focused on its acute effects within a short intervention duration. Few studies explored the longer-term effects of repeated sessions of CT (Berriel et al., 2022).

Blood flow restriction training (BFRT) is a novel strategy that can reduce risk of joint and muscle strains due to its integration of pressurized cuffs and low-load exercises (Lorenz et al., 2021). Recent studies have shown that interventions combining BFRT with other training methods (e.g., with HLRT) can induce improvements in athletic performance (e.g., jump height and sprint performance) that are at least comparable to HLRT in CT protocol (Yasuda et al., 2011; Hecht et al., 2016). However, the effects of an intervention CT combining BFR with the power of lower extremities (i.e., power output and bar velocity) have not been well-characterized (Wilk et al., 2022).

Therefore, in this pilot randomized and controlled study, we aimed to investigate the effects of 8-week CT with BFR on power outcomes. We hypothesized that CT with BFR would induce a significantly greater increase in power performance (e.g., one-repetition maximum squat test, vertical jump test, power output, and bar velocity) compared with CT-only protocol.

## 2 Materials and methods

### 2.1 Participants

Twenty-six healthy male university athletes were recruited for the study. Participants were randomized into group of CT_BFRT (age: 19.46 ± 0.83 years, height: 180.66 ± 3.67 cm, weight: 70.14 ± 7.58 kg, n = 13) and group of CT (age: 19.35 ± 0.73 years, height: 180.70 ± 6.17 cm, weight: 71.57 ± 6.78 kg, n = 13) (Table 1). The sample size of participants (i.e., n = 26) was determined using G-Power (version 3.1.9.7; Franz Faul, University of Kiel, Kiel, Germany) by using α err prob = 0.05; 1-β Err Prob = 0.8; effect size f = 0.4; test family = F test. The inclusion criteria were: (1) no experience in BFRT, but with experience in resistance training and plyometric training; (2) the ability to complete 1RM of squat test, jump test, and half-squat jump with linear position sensor; and; (3) commitment to complete the eight-week intervention and tests. The exclusion criteria were: (1) no anterior cruciate ligament (ACL), hamstring, meniscus, ankle, or other lower-extremity injuries that may affect their training and performance during BFRT intervention; and (2) any discomfort during BFRT intervention. The Research Ethics Committee approved the study protocol of Beijing Sport University (Approval number: 2022213H), and all procedures were conducted according to the Declaration of Helsinki. Before the experiment, participants were informed of the benefits and potential risks related to the study, and all signed the informed consent form.

**TABLE 1:** Physical characteristics of the subjects and 1RM of squat.

	Age (yrs)	Height (cm)	Weight (kg)	1RM of squat (kg)
CT_BFRT (n = 13)	19.46 ± 0.83	180.66 ± 3.67	70.14 ± 7.58	129.23 ± 11.15
CT (n = 13)	19.35 ± 0.73	180.70 ± 6.17	71.57 ± 6.78	128.46 ± 10.49

### 2.2 Experimental protocols

The participants completed three sessions of CT_BFRT or CT per week over 8 weeks at the gym of Beijing Sport University from January to April 2023. Details of CT_BFRT and CT programs can be found in Tables 2, 3. The CT_BFRT group had two 4-week phases: the first 4 weeks with cuff pressure set at 200 mmHg and the subsequent 4 weeks at 220 mmHg. Each session included four types of exercise (i.e., squat, split squat, deadlift, and subsequent jumps in the same manner). The load of the CT_BFRT group was at 20%–30% of the one-repetition maximum (1RM) of bilateral back squats, while CT group used 75%–80% of their 1RM of squats (Lixandrão et al., 2018). In the CT_BFRT group, all exercises were performed under lower limb occlusion with cuffs placed on the upper thighs at 30% of 1RM for squats (15–20 reps) and weighted half-squat jumps (6-8 reps) (Liu et al., 2022). CT group followed the same protocol without BFR. A total of three sets of a session, 4-min rest was provided between sets.

**TABLE 2:** Complex training with blood flow restriction program.

Complex pair	Intensity		Sets* repetitions	Rest (min)
	The first stage (1–4 weeks)	The second stage (5–8 weeks)		
Squat/Half-squat jump	20%1RM + Bar (200 mmHg)	30%1RM + Bar (220 mmHg)	3* (15–20 + 6–8)	4
Split squat/Split squat jump	20%1RM + ME (200 mmHg)	30%1RM + ME (220 mmHg)	3* (15–20 + 6–8)	4
Deadlift/Squat jump	20%1RM + ME (200 mmHg)	30%1RM + ME (220 mmHg)	3* (15–20 + 6–8)	4

Note: 1RM, 1-repetition maximum; ME, maximal effort.

**TABLE 3:** Complex training program protocol.

Complex pair	Intensity		Sets* repetitions	Rest (min)
	The first stage (1–4 weeks)	The second stage (5–8 weeks)		
Squat/Half-squat jump	75%1RM + Bar	80%1RM + Bar	4* (6–8 + 10–12)	4
Split squat/Split squat jump	75%1RM + ME	80%1RM + ME	4* (6–8 + 10–12)	4
Deadlift/Squat jump	75%1RM + ME	80%1RM + ME	4* (6–8 + 10–12)	4

Note: 1RM, 1-repetition maximum; ME: maximal effort.

All experimental training programs were conducted along with a weekly training routine and regular routine diet. Participants were prohibited from consuming beverages containing caffeine or alcohol throughout the intervention.

Before the initiation of the study, all participants completed a two-week familiarization (three sessions per week) with the same training protocols as used in the following intervention in this study. During the intervention period, participants in CT_BFRT group completed the intervention half-squat jump and plyometric training programs (Tables 2, 3) with BFR cuffs (B-strong, Alter G, United States of America), and participants in CT group completed the same training program without BFR cuffs (Cerqueira et al., 2016). Cuff pressure for training was set to 200 mmHg for the first 4-week intervention and 220 mmHg for the second 4-week intervention. Specifically, they completed three sessions of CT_BFRT for 8 weeks (i.e., twenty-four sessions). Considering that the physical status of participants on each training day may differ, we set a range of the number of required repetitions of the movement, which can make the task load similar to each subject. Within the training program, participants were asked first to perform squat with 30% 1RM for 15–20 repetitions (reps) and then the plyometric movement (half-squat jump) for 10–12 reps (Fernandez-Fernandez et al., 2016). The rest interval between CT_BFRT and plyometric exercise was 5–6 min. The rest between each set and exercise was 4 minutes. Two groups completed a standardized 8–15 min warm-up before every training session. The warm-up protocol included low-intensity running, coordination exercises, dynamic stretch movements, movement integration, and neural activation. After the training session, both groups performed a standard 8–15 min cooldown of static stretching. All participants were tested 3 days before and within 3 days after the intervention, and the test sequence, personnel, and location were consistent. All participants completed all the tests before the baseline and the baseline test 72 h later to assess the test-retest reliability.

Throughout each session, participants received consistent guidance and instruction from certified strength and conditioning coaches regarding correctly executing resistance and plyometric exercises. All protocols were meticulously designed and closely supervised by the research personnel, who were highly experienced researchers in strength and conditioning and fitness training.

### 2.3 Test procedure

Before and immediately after CT_BFRT and CT, the power accessed by one-repetition maximum squat testing, squat jump (SJ), countermovement jump (CMJ), and power output and bar velocity during half*-*squat jump with gymaware linear position sensor test as assessed by mean power (MP), peak power (PP), mean bar velocity (Bar-MV), and peak bar velocity (Bar-PV).

#### 2.3.1 One-repetition maximum squat testing

Lower limb strength was assessed with a 1RM squat, as reported by previous studies (Keiner et al., 2013; Jurado-Castro et al., 2022). The maximal load of the parallel back-squat exercise (1RM) was determined using procedures outlined by the National Strength and Conditioning Association (NSCA) (Miller, 2012). The movement for the parallel back-squat exercise was performed as described above for the squat training. Before 1RM measurement, the participants used 20 kg for 10 repetitions. Then, 50% estimated 1RM for five repetitions. After that, 75% estimated 1RM for three repetitions. Afterward, 90% estimated 1RM for one repetition to ensure maximal effort. Finally, the load increased by 5–10 kg and participants performed only one repetition until failure. The 1RM was typically determined within five to six trials. They were provided 3 min of rest between sets, which was considered sufficient.

#### 2.3.2 Countermovement jump test

The countermovement jump (CMJ) was used to assess lower limb power. During the CMJ test, participants were instructed to stand with their feet shoulder-width apart and keep their hands on their hips to prevent arm swings from impacting their jump height (Nonnato et al., 2022). They were then asked to quickly lower themselves into a squatting position of approximately 60°, then immediately jump as high as they can, and land in the same position as takeoff. The height achieved during each jump was recorded using a force platform (Kistler 9281CA, KISTLER, Winterthur, Switzerland). For tests, participants completed three maximal jumps with a 30-s rest period between trials. The maximum jump height was used in the analysis.

#### 2.3.3 Squat jump test

In the squat jump (SJ) test, participants first jumped from a semi-squat position with their hands still on their hips. They were then directed to bend their knees to about a 120-degree angle before jumping fully, attempting to avoid any countermovement, and to pause for 2 seconds at each phase (Comfort et al., 2012). Participants performed three maximal jumps for both conditions with a 30-s recovery time between trials and jumps. The take-off duration of each jump was monitored to ensure no preliminary steps or movements. The force platform was used to record take-off and landing time, and thus the duration of the flight phase. The calculation of SJ height was then completed using the equation proposed by Bosco (Keiner et al., 2013). The highest height was used for analysis.

#### 2.3.4 Half-squat jump with gymaware linear position sensor test

The lower limb power output was measured using the GymAware Power Tool linear position sensor (GymAware, Kinetic Performance Technology, Canberra, Australia). The mean power, peak power, peak velocity, and mean velocity were monitored and recorded during three load-bearing half-squat jumps (using a 20 kg empty barbell bar). At the beginning of each trial, participants were asked to maintain their upper body upright, keep their feet apart at shoulder width, and grasp the handles of the barbell bar with the Smith Machine. Next, they were asked to maintain their upper trunk stable, gradually lower by bending their knees and hip joints with their back straight, and fully extend their hips, knees, and ankles while in a half-squat. Then they rapidly jumped and landed back to the initial position. The maximum value of load-bearing half-squat jumps was obtained after three repetitions (Dorrell et al., 2019).

### 2.4 Statistical analyses

The experimental data were processed by the IBM SPSS statistical package (version 25.0, IBM Statistics, Chicago, IL, United States of America). Data were expressed as mean ± standard deviation (M ± SD). The normality of the data was assessed by the Shapiro-Wilk test. If the data was normally distributed, the differences in the demographics (i.e., age, weight, height, and 1RM of squat) and outcomes (i.e., SJ, CMJ, MP, PP, Bar-MV, and Bar-PV) at baseline were examined using one-way ANOVA.

If the data was normally distributed, we used two-way repeated-measure ANOVA to examine the effects of the intervention on the primary outcomes of 1RM of squat, SJ, and CMJ. The dependent variable for each model was each of the primary outcomes. The model effects were group (CT_BFRT and CT), time (pre- and post-intervention), and their interaction. When a significant interaction was observed, an LSD *post hoc* comparison was performed to identify where the significance was. Similar ANOVA models were used for secondary outcomes, including MP, PP, Bar-MV, and Bar-PV. We also conducted an exploratory analysis to examine the effects of time (i.e., pre- and post-intervention) on the outcomes within CT_BFRT and CT group using separate paired *t*-test models. Cohen’s d (d) values were used to assess the effect size, and it was classified as trivial (d < 0.2), small (0.2 ≤ d ≤ 0.6), moderate (0.6 ≤ d ≤ 1.2), large (1.2 ≤ d ≤ 2.0), or very large (d > 2.0) (Cohen, 2013). The significance level of these models was set at *p* < 0.05.

## 3 Results

All the participants completed this study, and all the data were included in the analysis. All the data were normally distributed (*p* > 0.14). No significant difference in the demographics (i.e., age, body weight, height, and 1RM of squat), primary outcomes measured (i.e., 1RM of squat, SJ, and CMJ), and secondary outcomes (i.e., MP, PP, Bar-MV, and Bar-PV) were observed between CT_BFRT and CT group (*p* > 0. 57) (Table 4).

**TABLE 4:** The assessment results for CT_BFRT group and CT group before and after 8-week training.

	CT_BFRT (N = 13)			Paired *t*-Test		CT (N = 13)			Paired *t*-Test		ANOVA (group x time)	
Variable	Pre	Post	Δ	*P*	Cohen’s d	Pre	Post	Δ	*P*	Cohen’s d	*P*	Cohen’s d
Squat 1RM (kg)	129.23 ± 11.15	137.31 ± 8.07	8.08 ± 4.35	<0.001	0.830	128.46 ± 10.49	138.85 ± 8.45	10.39 ± 3.80	<0.001	1.091	0.668	0.186
SJ (cm)	45.04 ± 3.02	51.16 ± 3.40	6.12 ± 1.57	<0.001	1.903	45.59 ± 4.57	50.32 ± 4.01	4.73 ± 1.84	<0.001	1.100	0.511	0.226
CMJ (cm)	48.05 ± 3.37	54.23 ± 2.6	6.19 ± 2.82	<0.001	2.053	48.34 ± 3.58	53.03 ± 2.33	4.68 ± 2.23	<0.001	1.553	0.374	0.486
MP (w)	37.69 ± 2.22	48.00 ± 5.58	10.30 ± 5.48	<0.001	2.428	36.67 ± 6.00	41.05 ± 4.38	4.38 ± 2.43	<0.001	0.834	0.030	1.386
PP (w)	67.20 ± 9.11	80.85 ± 7.95	12.86 ± 11.57	0.010	1.596	68.54 ± 9.32	71.42 ± 8.64	2.88 ± 2.94	0.004	0.320	0.032	1.136
Bar-MV (m/s)	1.73 ± 0.12	1.94 ± 0.11	0.20 ± 0.03	<0.001	1.824	1.72 ± 0.16	1.76 ± 0.17	0.04 ± 0.04	0.003	0.242	0.049	1.257
Bar-PV (m/s)	2.90 ± 0.25	3.26 ± 0.19	0.32 ± 0.33	0.024	1.621	2.92 ± 0.18	3.00 ± 0.14	0.08 ± 0.18	0.140	0.496	0.012	1.558

Δ changes between pre- and post-test.

**FIGURE 1:**
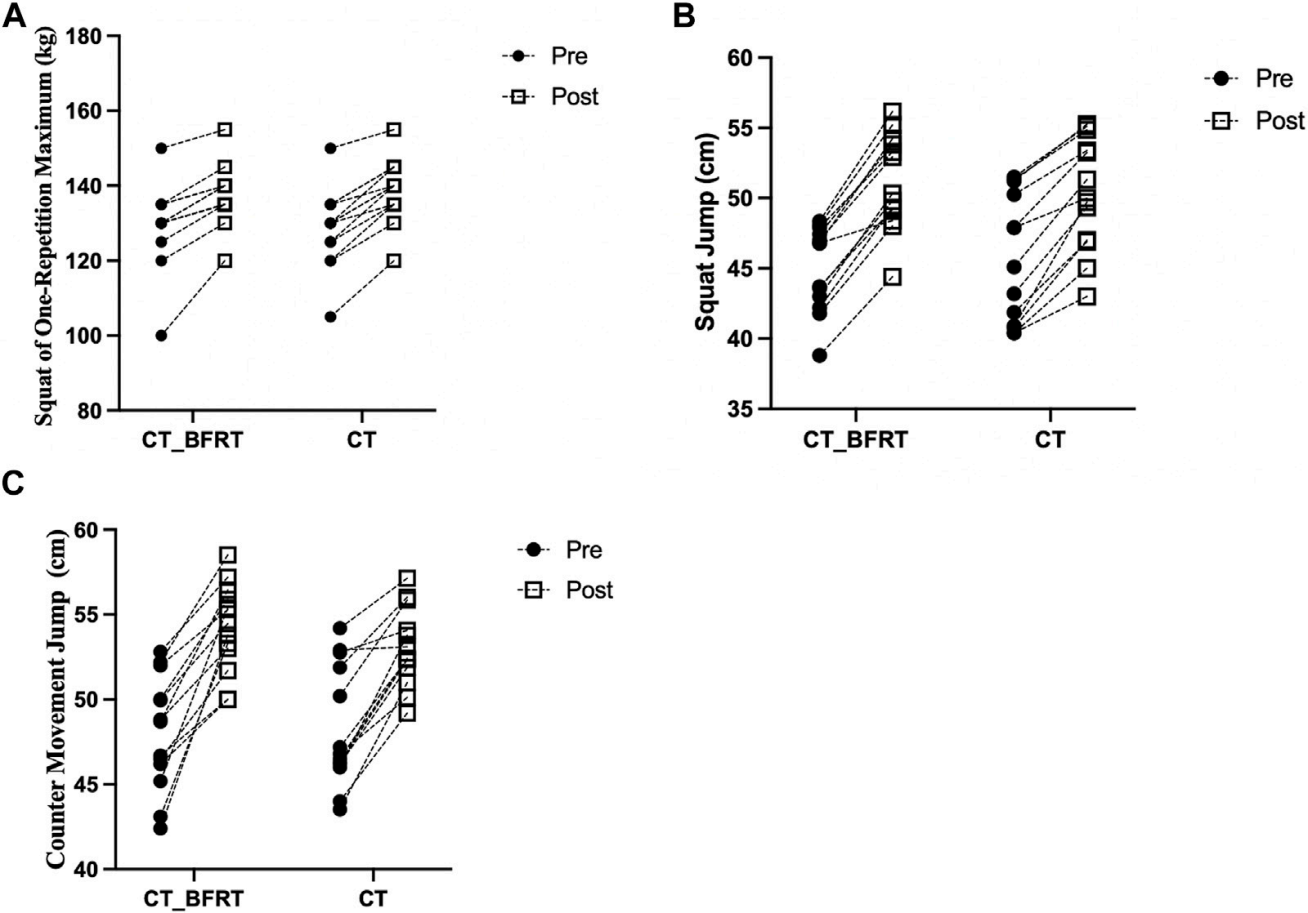
The 1R M of the squat **(A)**, SJ **(B)**, and CMJ **(C)** before and after intervention in CT_BFRT (n = 13) and CT-only (n = 13) group. Each dot on the figure represented each participant. Pre = Pre-exercise; Post = after intervention.

The primary two-way repeated-measures ANOVA models showed significant main effects of time (*p* < 0.001), but not group (*p* > 0.89) or interactions between group and time on 1RM of squat (*p* = 0.67, Figure 1A), SJ (*p* = 0.51, Figure 1B), or CMJ (*p* = 0.37, Figure 1C). The exploratory paired *t*-test models showed that within CT_BFRT group, 1RM of squat (*p* < 0.001, Cohen’s d = 0.83), SJ (*p* < 0.001, Cohen’s d = 1.90), and CMJ (*p* < 0.001, Cohen’s d = 2.05) were significantly improved after CT_BFRT as compared to baseline; and within CT group, 1RM of squat (*p* < 0.001, Cohen’s d = 1.10), SJ (*p* < 0.001, Cohen’s d = 1.10), and CMJ (*p* < 0.001, Cohen’s d = 1.55) were significantly improved after CT as compared to baseline.

**FIGURE 2:**
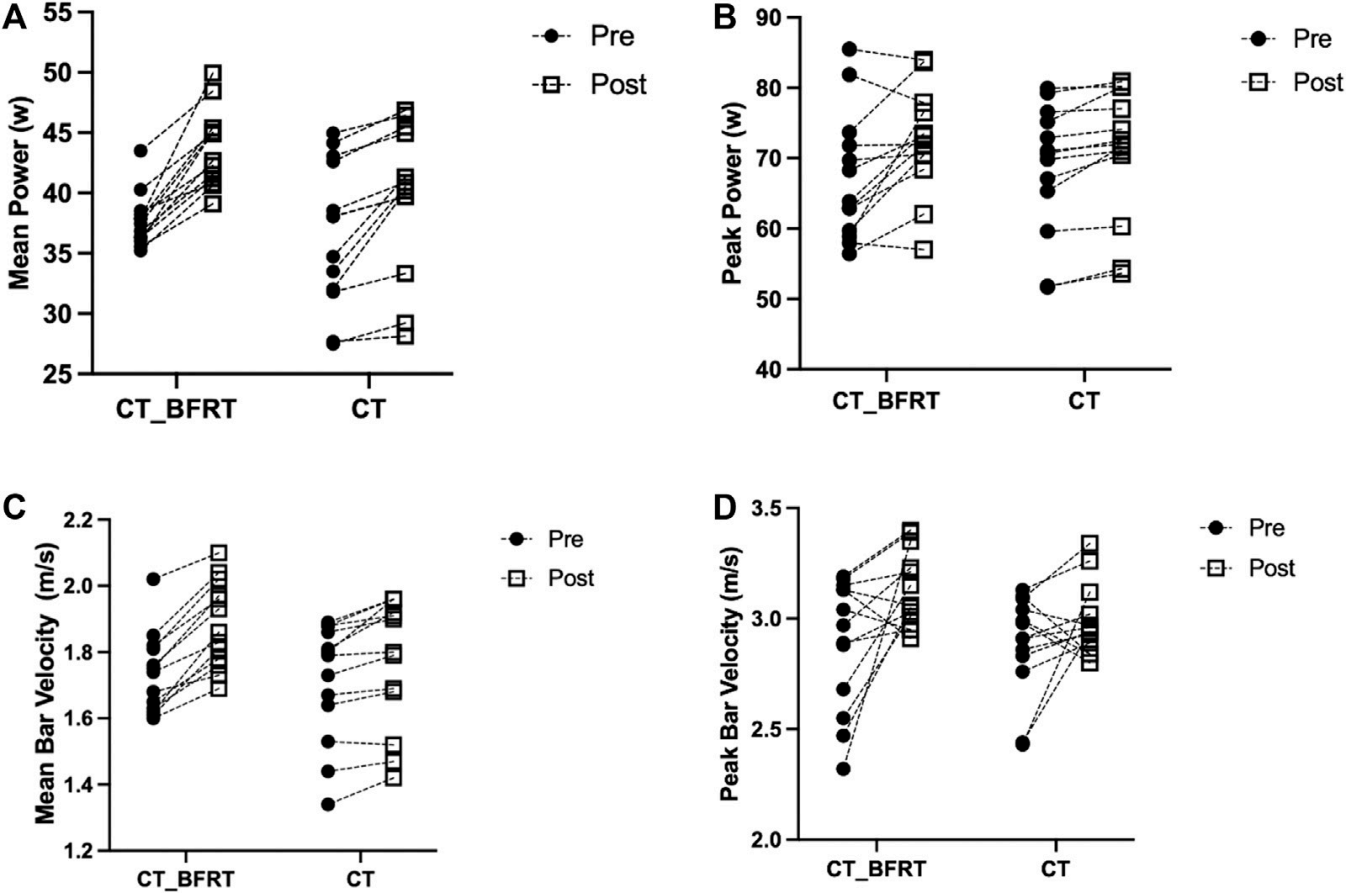
The MP **(A)**, PP **(B)**, Bar-MV **(C)**, and Bar-PV **(D)** before and after intervention in CT_BFRT (n = 13) and CT-only (n = 13) group. Each dot on the figure represented each participant. Pre = Pre-exercise; Post = after intervention.

The secondary two-way repeated-measures ANOVA models showed significant interactions between group and time on MP (*p* = 0.03, Cohen’s d = 1.39, Figure 2A), PP (*p* = 0.03, Cohen’s d = 1.14, Figure 2B), Bar-MV (*p* = 0.049, Cohen’s d = 1.26, Figure 2C), and Bar-PV (*p* = 0.01, Cohen’s d = 1.56, Figure 2D). The *post hoc* analysis revealed that MP [F (1,48) = 30.21, *p* < 0.001)], PP [F (1,48) = 15.74, *p* < 0.001], Bar-MV [F (1,48) = 12.94, *p* < 0.001] and Bar-PV [F (1,48) = 22.21, *p* < 0.001] were significantly greater after the CT with BFRT compared to all the other pre- and post-intervention conditions. The exploratory paired *t*-test analysis showed that within CT_BFRT group, MP (*p* < 0.001, Cohen’s d = 2.43), PP (*p* = 0.01, Cohen’s d = 1.60), Bar-MV (*p* < 0.001, Cohen’s d = 1.82) and Bar-PV (*p* = 0.02, Cohen’s d = 1.62) were significantly improved after CT_ BFRT as compared to baseline; and within CT group, MP (*p* < 0.001, Cohen’s d = 0.83), PP (*p* = 0.004, Cohen’s d = 0.32), and Bar-MV (*p* = 0.003, Cohen’s d = 0.24), but not Bar-PV (*p* = 0.14, Cohen’s d = 0.50), were significantly improved after CT only as compared to baseline.

## 4 Discussion

In this pilot study, we provide novel evidence of the effects of a CT combined with BFR on power output and bar velocity. The results here demonstrated that compared to complex training (CT) only, CT with blood flow restriction (CT_BFRT) induced comparable improvements in 1RM of squat, SJ, and CMJ of power, and significantly increased greater power output and bar velocity (i.e., MP, PP, Bar-MV, and Bar-PV) during half-squat jump. These observations suggest that this type of combined training, which consists of CT and BFRT, can more effectively improve power output and bar velocity than the CT-only protocol.

We observed no significant greater improvements in 1RM of squat, CMJ, and SJ of power induced by CT_BFRT compared to CT, suggesting CT_BFRT induced comparable benefits for power to CT-only protocol. One study demonstrated that combining 30% 1RM of CT with BFR induced no statistically significant decrease in subsequent squat jump height in participants. Such insignificance highlighted the potential influence of the CT protocol, rather than the loading intensity, on power outcomes of jump (Cleary and Cook, 2020). Our study was the first to demonstrate the effectiveness of repeated sessions of CT combined with BFR. Previous research reported that CT protocol included heavy-load slow-strength training and low-load fast-reinforcement activities, and this combination (i.e., HLRT and PT) might stimulate recruitment of high-order motor units and excitability mechanisms, leading to increased power generation in subsequent movements (Tillin and Bishop, 2009; Liu et al., 2022). CT is primarily influenced by mechanical factors of HLRT, while BFRT can enhance metabolic stress and induce equivalent training adaptations to traditional resistance training (Krzysztofik et al., 2020). Research reported that using BFRT in combination with other training approaches may enhance power by increasing the activation of motor units at low loads as comparable to HLRT-only (Lixandrão et al., 2018). Our results may be attributed to equivalent training adaptability by CT_BFRT, which shows that CT with BFR produces similar enhancements in power-related outcomes compared to CT only. Notably, these observations indicate that CT_BFRT, which has lower physical load than CT, could be a strategy that can help present potential injuries due to high-load training.

We observed that power as assessed by power output and bar velocity during half-squat jump were significantly higher performed in CT_BFRT group as compared to CT group. The power output levels generated by muscles depend on substrates such as adenosine triphosphate (ATP) and metabolic mechanisms. BFRT may be a performance-enhancing stimulus during explosive resistance training (Wilk et al., 2021). BFRT during high-load resistance exercise may increase mechanical tension and metabolic stress, thus enhancing the effects of resistance training (Trybulski et al., 2021). This aligns with previous research on acute changes in power output and bar velocity during bench press with BFRT (Wilk et al., 2022). Our study suggests that multiple sessions of CT_BFRT have the potential to enhance the sustainability of power output and bar velocity in the half-squat jump. On the other hand, no significant changes were observed in CT-only group, potentially due to the absence of a metabolic stimulus, leading to a lack of potential synergistic effects on muscles. Additionally, improvement in performance induced by CT_BFRT may also be attributed to more effective utilization in neuromuscular adaptation of postactivation potentiation enhancement (PAPE) that BFRT induced greater fast muscle fiber recruitment (Poulos et al., 2023). As demonstrated in a previous study, muscle activation stimulates power output and bar velocity during consecutive sets of resistance exercises (Wilk et al., 2020). Our observation thus suggests that low-intensity CT combined with BFR may offer a promising advantage in power output and bar velocity in half-squat jump compared to CT-only protocol.

### 4.1 Limitations

This pilot study has several limitations that should be acknowledged. First, only twenty-six young males were included. Future studies consisting of a larger number of participants with a balanced number of sexes are needed to examine and confirm the observations in this study. Second, the recruitment of participants with experience in resistance training and plyometric training may potentially limit the study findings to special populations (e.g., athletes undergoing injury rehabilitation). Third, it is worthwhile to examine whether more easily controlled parameters (e.g., pressure levels and cuff widths) of this combined protocol may be feasible and beneficial for other populations with limited functions (e.g., people with less strength) compared to traditional CT protocol.

## 5 Conclusion

This study showed that complex training combined with blood flow restriction may induce significant greater improvements in power output and bar velocity during half-squat jump, and comparable improvements in 1RM of squat, CMJ, and SJ of power to the protocol of using complex training only.

## Data Availability

The original contributions presented in the study are included in the article/Supplementary Material, further inquiries can be directed to the corresponding authors.
