# Supplementary material for: Complex training with blood flow restriction increases power output and bar velocity during half-squat jump: a pilot randomized controlled study
Source: Front Physiol. 2024 May 30;15:1368917. doi: 10.3389/fphys.2024.1368917 (PMC11177751; doi:10.3389/fphys.2024.1368917)
Supplement: Supplementary file 1 [file DataSheet1.docx]

Supplementary Material

# Supplementary Figures and Tables

## Supplementary Figures
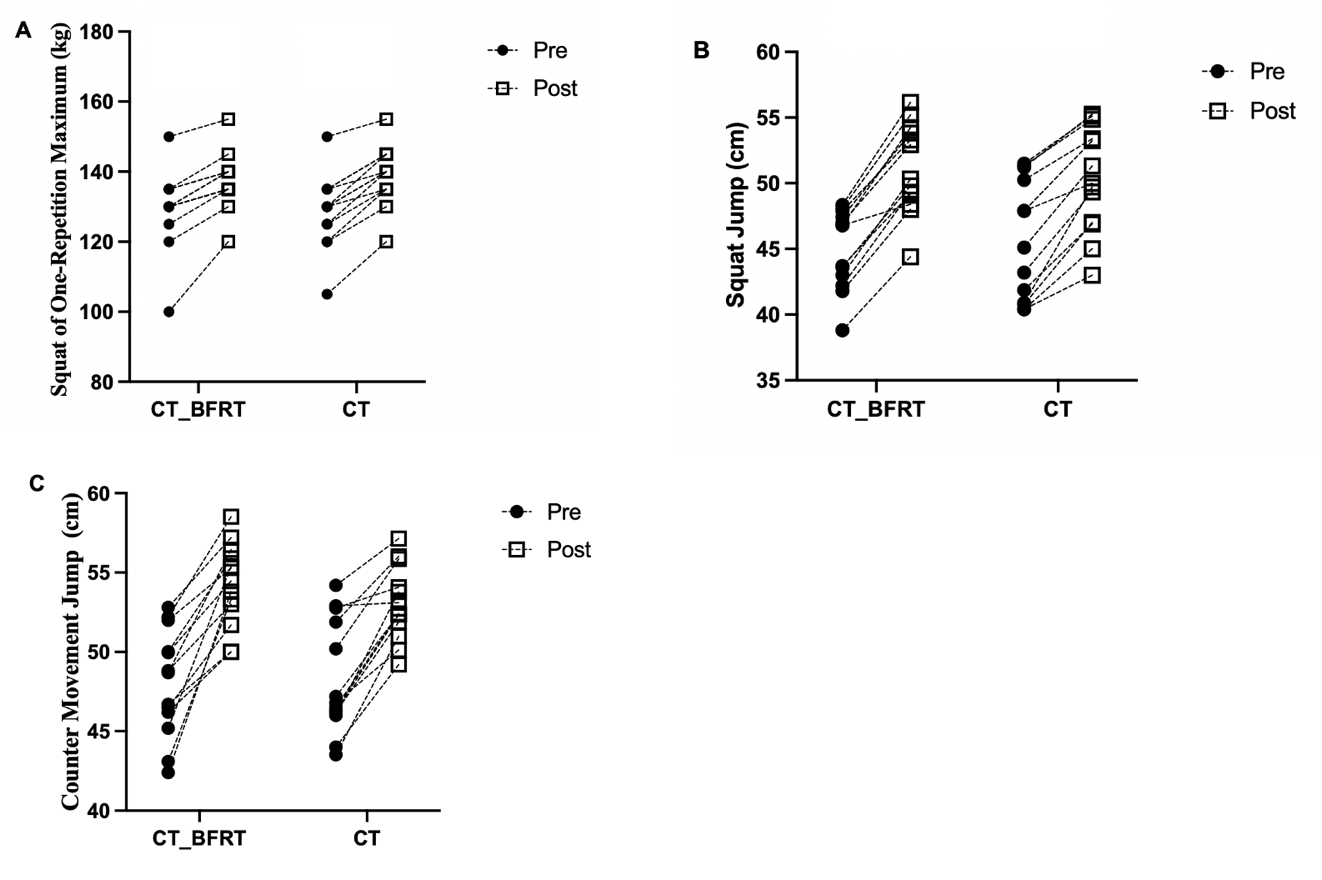


**Figure 1.** The 1RM of the squat (A), SJ (B), and CMJ (C) before and after intervention in CT_BFRT (n=13) group and CT-only (n=13) group. Each dot on the figure represented each participant. Pre = Pre-exercise; Post = after intervention.


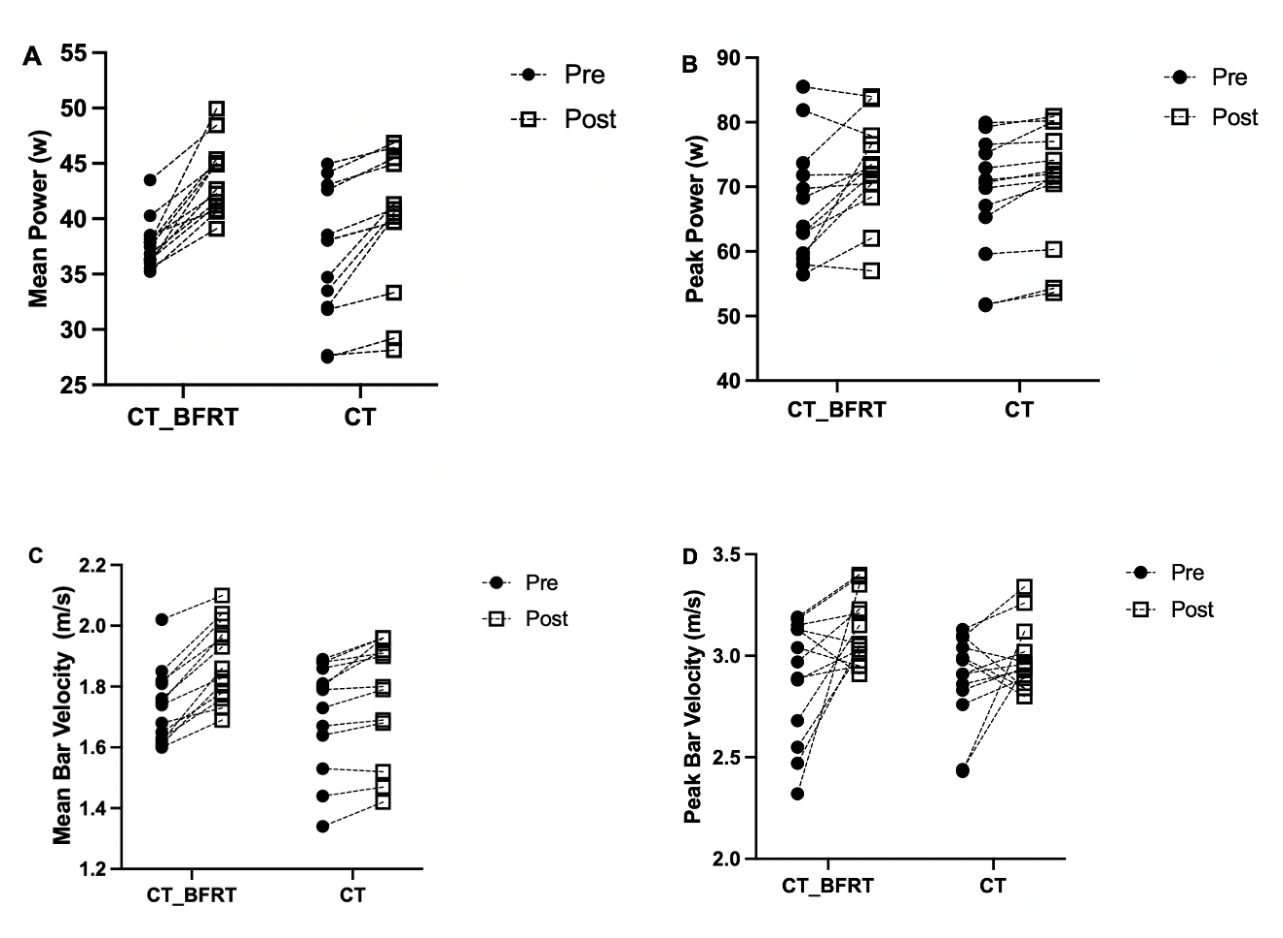


## Figure 2. The MP (A), PP (B), Bar-MV (C), and Bar-PV (D) before and after intervention in CT_BFRT (n=13) group and CT-only (n=13) group. Each dot on the figure represented each participant. Pre = Pre-exercise; Post = after intervention.

## Supplementary Tables

**Table 1.** Physical characteristics of the subjects and 1RM of squat

|  | Age (yrs) | Height (cm) | Weight (kg) | 1RM of squat (kg) |
| --- | --- | --- | --- | --- |
| CT_BFRT  (n = 13) | 19.46 ± 0.83 | 180.66 ± 3.67 | 70.14 ± 7.58 | 129.23 ± 11.15 |
| CT (n = 13) | 19.35 ± 0.73 | 180.70 ± 6.17 | 71.57 ± 6.78 | 128.46 ± 10.49 |

**Table 2.** Complex Training with Blood Flow Restriction Program

| **Complex pair** | **Intensity** | | **Sets* repetitions** | **Rest (min)** |
| --- | --- | --- | --- | --- |
|  | **The ﬁrst stage**  **(1–4 weeks)** | **The second stage (5–8 weeks)** |  |  |
| Squat / Half-squat jump | 20%1RM+Bar (200mmHg) | 30%1RM+Bar (220mmHg) | 3* (15～20+6～8) | 4min |
| Split squat / Split squat jump | 20%1RM+ME (200mmHg) | 30%1RM+ME (220mmHg) | 3* (15～20+6～8) | 4min |
| Deadlift / Squat jump | 20%1RM+ME (200mmHg) | 30%1RM+ME (220mmHg) | 3* (15～20+6～8) | 4min |

Note:1RM, 1-repetition maximum; ME, maximal effort.

**Table 3.** Complex Training Program Protocol

| **Complex pair** | **Intensity** | | **Sets* repetitions** | **Rest (min)** |
| --- | --- | --- | --- | --- |
|  | **The ﬁrst stage**  **(1–4 weeks)** | **The second stage (5–8 weeks)** |  |  |
| Squat / Half-squat jump | 75%1RM+Bar | 80%1RM+Bar | 4* (6～8+10～12) | 4min |
| Split squat / Split squat jump | 75%1RM+ME | 80%1RM+ME | 4* (6～8+10～12) | 4min |
| Deadlift / Squat jump | 75%1RM+ME | 80%1RM+ME | 4* (6～8+10～12) | 4min |

Note: 1RM, 1-repetition maximum; ME: maximal effort.

**Table 4．** The assessment results for CT_BFRT group and CT group before and after 8-week training

|  | **CT_BFRT (N = 13)** | | | **Paired t-Test** | | **CT (N = 13)** | | | **Paired t-Test** | | **ANOVA (Group x Time)** | |  |
| --- | --- | --- | --- | --- | --- | --- | --- | --- | --- | --- | --- | --- | --- |
| Variable | Pre | Post | Δ | *P* | Cohen’s d | Pre | Post | Δ | *P* | Cohen’s d | *P* | Cohen’s d |  |
| Squat 1RM (kg) | 129.23 ± 11.15 | 137.31 ± 8.07 | 8.08 ± 4.35 | < 0.001 | 0.830 | 128.46 ± 10.49 | 138.85 ± 8.45 | 10.39 ± 3.80 | < 0.001 | 1.091 | 0.668 | 0.186 |  |
| SJ (cm) | 45.04 ± 3.02 | 51.16 ± 3.40 | 6.12 ± 1.57 | < 0.001 | 1.903 | 45.59 ± 4.57 | 50.32 ± 4.01 | 4.73 ± 1.84 | < 0.001 | 1.100 | 0.511 | 0.226 |  |
| CMJ (cm) | 48.05 ± 3.37 | 54.23 ± 2.6 | 6.19 ± 2.82 | < 0.001 | 2.053 | 48.34 ± 3.58 | 53.03 ± 2.33 | 4.68 ± 2.23 | < 0.001 | 1.553 | 0.374 | 0.486 |  |
| MP (w) | 37.69 ± 2.22 | 48.00 ± 5.58 | 10.30 ± 5.48 | < 0.001 | 2.428 | 36.67 ± 6.00 | 41.05 ± 4.38 | 4.38 ± 2.43 | < 0.001 | 0.834 | 0.030 | 1.386 |  |
| PP (w) | 67.20 ± 9.11 | 80.85 ± 7.95 | 12.86 ± 11.57 | 0.010 | 1.596 | 68.54 ± 9.32 | 71.42 ± 8.64 | 2.88 ± 2.94 | 0.004 | 0.320 | 0.032 | 1.136 |  |
| Bar-MV (m/s) | 1.73 ± 0.12 | 1.94 ± 0.11 | 0.20 ± 0.03 | < 0.001 | 1.824 | 1.72 ± 0.16 | 1.76 ± 0.17 | 0.04 ± 0.04 | 0.003 | 0.242 | 0.049 | 1.257 |  |
| Bar-PV (m/s) | 2.90 ± 0.25 | 3.26 ± 0.19 | 0.32 ± 0.33 | 0.024 | 1.621 | 2.92 ± 0.18 | 3.00 ± 0.14 | 0.08 ± 0.18 | 0.140 | 0.496 | 0.012 | 1.558 |  |
| Δ changes between pre- and post- test. | | | | | | | | | | | | |  |
